# The Relationship between Dioxin Congeners in the Breast Milk of Vietnamese Women and Sister Chromatid Exchange

**DOI:** 10.3390/ijms15057485

**Published:** 2014-04-30

**Authors:** Hiroyuki Suzuki, Teruhiko Kido, Rie Okamoto, Dang Duc Nhu, Muneko Nishijo, Hideaki Nakagawa, Kenji Tawara, Hiroaki Horikawa, Yuko Sato, Phung Tri Dung, Le Hong Thom, Nguyen Ngoc Hung

**Affiliations:** 1 Department of Nursing, Toyota West Hospital, 100 Yokoyama, Homi-cho, Toyota, Aichi 470-0344, Japan; E-Mail: hirobocckle@hotmail.com; 2 Faculty of Health Science, Institute of Medical, Pharmaceutical and Health Sciences, Kanazawa University, 5-11-80 Kodatsuno, Kanazawa, Ishikawa 920-0942, Japan; E-Mail: naganuma@mhs.mp.kanazawa-u.ac.jp; 3 School of Medicine and Pharmacy, Vietnam National University, 144 Xuan Thuy Str., Cau Giay Dist., Hanoi 100803, Vietnam; E-Mail: dangnhu258@yahoo.com; 4 Department of Epidemiology and Public Health, Kanazawa Medical University, 1-1 Daigaku, Uchinada, Kahoku, Ishikawa 920-0293, Japan; E-Mails: ni-koei@kanazawa-med.ac.jp (M.N.); hnakagaw@kanazawa-med.ac.jp (H.N.); 5 Department of Environment Technology and Measurement, Hyogo Environmental Advancement Association, 3-1-31 Yukihira-cho, Suma-ku, Kobe, Hyogo 654-0037, Japan; E-Mail: tawaran500@gmail.com; 6 Division of Ultrafine Structure, Department of Pathology, Research Institute, International Medical Center of Japan, 1-21-1 Toyama, Shinjuku, Tokyo 162-8655, Japan; E-Mails: amigo825hiro@goo.jp (H.H.); ysato@ri.imcj.go.jp (Y.S.); 7 Centre for Environment and Population Health, Griffith School of Environment, Griffith University, 170 Kessels Road, Nathan Campus, Brisbane, QLD 4111, Australia; E-Mail: d.phung@griffith.edu.au; 8 Division for Mitigation of the Consequences of the Chemicals used during the War on Human Health (10-80 Division), Hanoi Medical University, 1 Ton That Tung Str., Dong Da Dist., Hanoi 100803, Vietnam; E-Mails: hongthomle@yahoo.com (L.H.T.); hungdhyhn@gmail.com (N.N.H.)

**Keywords:** dioxins, furans, congener, sister chromatid exchange, Vietnam

## Abstract

The aim of this study was to clarify the relationship between dioxin concentrations in breast milk and the sister chromatid exchange (SCE) frequency in women from herbicide-sprayed and non sprayed areas. Blood samples were taken from 21 women with high TCDD (tetrachlorodibenzo-p-dioxin) levels from sprayed areas, 23 women with moderate TCDD levels from sprayed areas, and 19 women from non sprayed areas to determine their SCE frequency. The SCE frequencies for the high and moderate TCDD groups from the sprayed area and for the non sprayed area group were 2.40, 2.19, and 1.48 per cell, respectively. Multiple regression analysis showed that the standardized β values for 1,2,3,6,7,8-hexaCDD (β = 0.60), 1,2,3,4,6,7,8-heptaCDD (β = 0.64), and octaCDD (β = 0.65) were higher than those for TCDD (β = 0.34) and 1,2,3,7,8-pentaCDD (β = 0.42). The adjusted *R*^2^ value for polyCDDs (*R*^2^ = 0.38) was higher than that for polyCDD toxic equivalents (TEQ (toxic equivalents); *R*^2^ = 0.23). This study therefore shows that levels of hexa-, hepta-, and octaCDD, which were previously regarded as being less toxic than TCDD, are closely related to SCE frequency and that the level of dioxin (pg/g lipid) is potentially more useful as an indicator than TEQ value for explaining SCE frequency.

## 1. Introduction

During the Vietnam War (1961–1972), the U.S. military sprayed herbicide over large areas of southern Vietnam to destroy forests and crops in an operation known as Ranch Hand. However, the primary mixture of organochlorine herbicides used, known as Agent Orange, was contaminated with dioxins. The spray inventory with heavily dioxin-contaminated herbicides has been estimated at more than 76.5 million liters [1].

Several studies investigating the health effects of these herbicides in American and Korean subjects have been published [2–5], whereas, for Vietnamese subjects, dioxin concentrations in breast milk have been analyzed by pooling samples [6,7]. However, these dioxin studies focused on 2,3,7,8-tetrachlorodibenzo-p-dioxin (TCDD) and did not consider other congeners [8,9].

In light of this, we initiated a study in 2002 that involved collecting breast milk from individual lactating mothers living in herbicide-sprayed and non sprayed areas. The results of this study showed that the dioxin concentrations in breast milk and soil samples obtained from the sprayed area are still much higher than those from non sprayed areas [10]. As a result of these findings, we subsequently investigated the relationship between dioxin concentrations in breast milk and food group intake [11] and soil or sediment samples using a geographic information system [12].

Xinh *et al*. [13] have reported a much shorter survival time in chronic myeloid leukemia patients from Southern Vietnam as a result of pre-existing genetic instabilities that induce various secondary chromosome abnormalities and multiple clones, although it is not known whether dioxin exposure is responsible for these instabilities.

Previous studies concerning the genetic effects of dioxin have investigated sister chromatid exchange (SCE) frequency, which is a widely adopted and sensitive tool for measuring genetic damage caused by carcinogens or mutagens as it considered to reflect damage to DNA repair systems [14]. Landgren *et al.* [15], for example, found a significant correlation between α-naphthoflavone (ANF)-induced SCEs and serum concentrations of Polychlorinated Biphenyls (PCBs) and several PCB congeners in women from Yucheng. Likewise, the SCE frequency for New Zealand Vietnam veterans has been found to be higher than that for the control group [16].

Dioxins are considered to express their toxicities via aryl hydrocarbon receptor (AHR) pathway [17], and exposure to dioxins leads to negative feedback by expression of AHR repressor (AHRR) that inhibits AHR pathway [18]. Some of us [19] have reported a positive association between internal total toxic equivalents (TEQ) in breast milk and SCE frequency in herbicide-sprayed and non sprayed areas in Vietnam, and this observation might be explained by negative feedback effect by induced AHRR expression.

Although animal studies [20,21] or *in vitro* studies [22,23] have proved that exposure to dioxins increases SCE values, no studies have shown the correlation between internal dioxins levels and SCE values on humans. Even more dioxin congeners were not considered. The purpose of this study is therefore to clarify the association between SCE frequency and several dioxin congeners.

## 2. Results and Discussion

### 2.1. Characteristics

The mean monthly family income for the high TCDD group was significantly (*p* < 0.05) higher than that for the non sprayed group (1,500,000 and 800,000 Vietnamese Dong (VND), respectively; see Table 1), and there were significantly fewer farmers were in the high TCDD group (*p* < 0.01) than in the moderate TCDD group (52.4% and 95.7%, respectively; see Table 1). Job category “other” consisted of three farmer with a side jobs (worker, business and trade), two business, two teachers, one trade, one tailor, and one no response in the high TCDD group and one farmer with a side jobs (business) in the moderate TCDD group, and two farmer with a side jobs (fisher and fish farming) and one teacher in the non sprayed group. Other characteristics were not statistically significant.

### 2.2. SCE (Sister Chromatid Exchange) Frequency and Dioxin Concentration

Sister chromatid exchange frequencies were calculated for an average of 35.7 cells (range: 20–48) per individual for 44 individuals in the sprayed area and 19 in the non sprayed area. The SCE frequency for the high TCDD group (2.40 per cell) was significantly higher than those for the moderate TCDD (2.19 per cell) and non sprayed groups (1.48 per cell; *p* < 0.05 and *p* < 0.001, respectively). Likewise, the SCE frequency for the moderate TCDD group was significantly higher (*p* < 0.001) than that for the non sprayed group (Table 2).

As regards dioxins, seven congeners were found in significantly higher concentration in the high TCDD group than in the other two groups (1,2,3,4,6,7,8-heptaCDD: high TCDD group *vs*. moderate TCDD group, *p* < 0.01; high TCDD group *vs*. non sprayed group, *p* < 0.001; octaCDD: high TCDD group *vs*. moderate TCDD group, *p* < 0.05; high TCDD group *vs*. non sprayed group, *p* < 0.001. Other congeners: high TCDD group *vs*. moderate TCDD group, *p* < 0.001; high TCDD group *vs.* non sprayed group, *p* < 0.001), and these congeners, except for TCDD, were also found in significantly higher amounts in the moderate TCDD group than in the non sprayed group (1,2,3,7,8-pentaCDD and 1,2,3,4,7,8-hexaCDD: *p* < 0.05. Other congeners: *p* < 0.001).

Nine furan congers were present in significantly higher concentrations in the high TCDD group than in the other two groups and were also present in significantly higher concentrations in the moderate TCDD group than in the non sprayed group. The only exception to this trend was 2,3,7,8-tetraCDF, which was present in significantly higher concentrations in the non sprayed group than in the other two groups.

### 2.3. Single Regression Analysis of SCE Frequency and Dioxin Congeners

**Figure 1.**
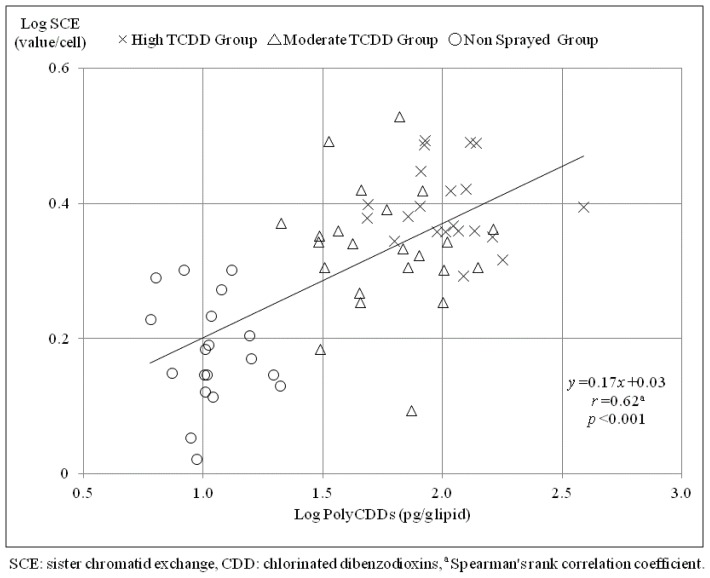
Comparison with the correlation between SCE and total polyCDDs.

Significant positive correlations were found between SCE frequency and seven dioxin congers (e.g., 1,2,3,6,7,8-hexaCDD: *r* = 0.63, *p* < 0.001; Table 3), whereas a significant negative correlation was found between SCE frequency and 2,3,7,8-tetraCDFs (*r* = −0.42, *p* < 0.001). A significant or non-significant positive correlation was found between SCE frequency and the nine furan congeners (e.g., 1,2,3,4,7,8,9-heptaCDF: *r* = 0.56, *p* < 0.001). Significant positive correlations were found between SCE frequency and each total measured value (e.g., polyCDDs: *r* = 0.62, *p* < 0.001; Figure 1) and each total-TEQ value (e.g., polyCDDs-TEQ: *r* = 0.56, *p* < 0.001).

### 2.4. Multiple Regression Analysis of SCE Frequency and Regional Difference

Categorical data for the sprayed area were employed as independent variables in model 1, and the high TCDD and non sprayed groups were employed as independent variables in model 2 (Table 4).

In model 1, taking the non sprayed area as reference category, the sprayed area was found to be positively associated with SCE frequency (β = 0.73, *R*^2^ = 0.49). Likewise, with model 2, when the moderate TCDD group was used as the reference category, the high TCDD group was positively, and the non sprayed group negatively, associated with SCE frequency (β = 0.30 and β = −0.58, respectively; *R*^2^ = 0.54).

### 2.5. Multiple Regression Analysis of SCE Frequency and Dioxin Congeners

Each dioxin congener and the total and total-TEQ value for polyCDDs, polyCDFs, and polyCDDs/Fs were employed as independent variables (Table 5).

All dioxin congeners were positively associated with SCE frequency, and the standardized β and adjusted *R*^2^ values for 1,2,3,6,7,8-hexaCDD (β = 0.60, *R*^2^ = 0.35), 1,2,3,7,8,9-hexaCDD (β = 0.48, *R*^2^ = 0.23), 1,2,3,4,6,7,8-heptaCDD (β = 0.64, *R*^2^ = 0.39) and octaCDD (β = 0.65, *R*^2^ = 0.37) were higher than those for TCDD (β = 0.34, *R*^2^ = 0.09), and 1,2,3,7,8-pentaCDD (β = 0.42, *R*^2^ = 0.16).

Six furan congeners, not including 1,2,3,7,8-pentaCDF, were positively associated with SCE frequency, and the standardized β and adjusted *R*^2^ values for 1,2,3,4,7,8-hexaCDF (β = 0.51, *R*^2^ = 0.26), 1,2,3,6,7,8- hexaCDF (β = 0.51, *R*^2^ = 0.26), 1,2,3,4,6,7,8-heptaCDF (β = 0.54, *R*^2^ = 0.29), and 1,2,3,4,7,8,9-heptaCDF (β = 0.58, *R*^2^ = 0.32) were also higher than those for TCDD and 1,2,3,7,8-pentaCDD.

With regard to total measured value and total-TEQ value, the standardized β and adjusted *R*^2^ values for polyCDDs (β = 0.64, *R*^2^ = 0.38) were higher than those for polyCDFs (β = 0.47, *R*^2^ = 0.22), and those for polyCDDs, polyCDFs, and polyCDDs/Fs were higher than those for each total-TEQ value (e.g., polyCDDs/Fs: β = 0.59, *R*^2^ = 0.32; polyCDDs/Fs-TEQ: β = 0.48, *R*^2^ = 0.21).

### 2.6. Discussion

Hexa-, hepta-, and octaCDD, along with hexa- and heptaCDF, which account for a large proportion of the dioxins found in Vietnamese women and which were previously regarded as being less toxic than TCDD, have, for the first time, been related to SCE frequency by using a multivariate analysis, adjusted for age, number of children, smoking status, and other confounding factors [24–27]. Nagayama *et al.* [25] shows that no relationship between SCE and 2,3,7,8-TCDD. This sample size is 39 healthy Japanese (25 male and 14 females, mean age: 44.3 years old and the range 20–64 years old). The previous study shows that correlation of 2,3,7,8-TCDD is lower than other dioxin congers and sampling method is different from this study (we use a stratified sampling method based on 2,3,7,8-TCDD concentrations).

With regard to confounding factors related increase in SCE. Nagayama *et al.* [25] shows increase of age related to both SCE and dioxins. The result of our study shows the family income and occupation are difference between three groups. Therefore, we adjusted those confounding factor (age, number of children, family income, occupation, and smoking status) by multiple regression analysis. But in this study did not concern effect from food. The high TCDD group in sprayed area included more high-income job such as business, teacher, *etc.*, than others. We supposed that people with higher income consume more meat than those with lower income. More investigation is needed.

Additionally, we consider about agricultural pesticide as one of confounding factors, which also induce SCE frequency [28]. We have already examined the consumption volume of pesticide and we found less consumption in sprayed area than non sprayed area (74 litters *vs.* 374 litters (/family/year)) Vietnam is a socialistic country and they have their own unique social system. Pesticide consumption is controlled by the people committee in each commune [29]. We could only obtain the data on total amount of pesticide in each commune and could not receive the individual consumption data unfortunately. Finally we consider that agricultural chemicals are less affected in sprayed area. However, this point is our study limitation.

Thus, the dioxin concentration has been shown to be potentially useful as an indicator than TEQ value for explaining the relationship with SCE frequency. These results may indicate that genetic damage due to dioxin exposure differs from the toxicity of TEFs [30].

On the analysis of SCE, the baseline SCE frequencies in this study are unusually low with most other studies in the literature showing SCE frequencies of from 5 to 8 SCEs/metaphase, not 1 to 3 has shown here. However, it depends on how to analyze SCE. The samples were then exposed to 1 μg/mL BrdU for 38 h and 20 ng/mL colcemid for 2 h in this study. But other literature, Rowland *et al.* [16] said that “0.01 M BrdU was added to culture tube, After approximately 72 h, 100 μL of 0.05% colchicines was added. Thus, it is important to consider the significantly different methods between those groups.

The number of cells scored/person is small in this study. Twenty–five is usually considered to be a minimum. We actually collected those blood samples from each area and transferred to Ho Chi Ming city by airplane, which takes half a day. It is difficult to analyze instantly through field-work, still more overseas developing county [31]. However, interquartile range is 27–42 and median is 40 cells per individual. Therefore, we believe that the results of SCE frequency were influence limited. However, in the present study, there are already limitations of subject numbers.

The subjects included were all second-generation and were, therefore, not directly exposed to herbicides. The Vietnam War ended more than 30 years ago; therefore, individual dioxin congener concentrations are decreasing at different rates due to their different half lives [32]. Their concentrations are, however, still higher than those in non sprayed areas. Dioxin concentration is currently not sufficient to describe the relationship with SCE frequency, as the *R*^2^ values adjusted for regional differences are much higher than those for dioxin concentrations. Saito *et al.* [11] suggested that dioxin concentrations in breast milk are not influenced by present dietary intake. We, therefore, suggest that long term exposure to the high dioxin levels found in the sprayed area causes SCE. Garaj-Vrhovac and Zeljezic [28] have also reported that previous long-term exposure to a mixture of pesticides affects SCE frequency.

In this study, we selected subjects based on TCDD concentration, although this was subsequently not found to be strongly related to SCE frequency. Selection by total polyCDD concentration potentially, however, shows a clearer relationship between SCE frequency and dioxin levels.

## 3. Experimental Section

### 3.1. Study Area and Population

The study area was the north-central area of Vietnam, namely Cam Chinh commune in the Cam Lo district of Quang Tri province, which was sprayed with herbicides during the war, and the Cam Phuc commune in the Cam Xuyen district of Ha Tinh province, which was not sprayed with herbicides. These two provinces were once separated by the demilitarized zone (DMZ) along the 17th parallel, which divided the country during the war. Quang Tri province was located to the south of the DMZ.

A stratified sampling method based on TCDD concentrations in breast milk collected from 20 to 30 years old lactating females in September 2002 and July 2003 were used in this study [10]. We set up three groups from 90 females in the sprayed area by systematic sampling based on TCDD concentrations (high, middle, and low groups; the latter 2 groups are combined as moderate) and selected 25 subjects from each of the high and moderate groups. For the non sprayed area, we selected a further group of 25 subjects from 74 females by systematic sampling, based on TCDD levels. Interviews were performed using a questionnaire to obtain personal information. The number of children was determined when breast milk samples were obtained in 2002 or 2003. This survey was performed in August 2007.

A lack of characteristic information was found in the sprayed area, and four people from the non sprayed area were unable to attend because of the large typhoon that passed over this area at that time. Finally, 24 subjects with high TCDD levels and 25 subjects with moderate TCDD levels from the sprayed area, along with 21 subjects from the non sprayed area, were included in this study.

### 3.2. Analysis of SCE

Peripheral blood samples were collected from all subjects (49 from the sprayed area and 21 from the non sprayed area). A portion of each sample (0.5 mL) was cultured in a bottle with 9.5 mL RPMI-1640 medium and 2% phytohaemagglutinin-M at 37 °C in a humidified atmosphere containing 5% CO_2_ for 34 h. The samples were then exposed to 1 μg/mL BrdU for 38 h and 20 ng/mL colcemid for 2 h. Cells were harvested and preparation slides were obtained using a standard method [33]. Fluorescence-plus Giemsa (FPG) staining was performed according to a previously reported procedure [34]. After confirming the existence of 46 complete chromosomes, consecutive second mitotic metaphase cells with differentially stained sister chromatids were analyzed for SCE frequency with the analyst being blinded to the corresponding dioxin levels.

Seven blood samples were found to be biologically contaminated, which meant that a total of 21 samples for the high TCDD group, 23 samples for the moderate TCDD group (both from the sprayed area) and 19 samples from the non sprayed area were included in this study. In addition, those SCE data were same as Horikawa *et al*. [19].

### 3.3. Analysis of Dioxin

Breast milk samples (10–20 mL) were collected from lactating primiparous and multiparous mothers and 10 mL of these were used for analysis of dioxin.

The fat content in breast milk was determined by the method reported by Patterson *et al.* [35]. The methodology, including cleanup steps and the separation and collection of the polychlorinated dibenzo-p-dioxins (polyCDDs) and polychlorinated dibenzofurans (polyCDFs) from fat, was described in our previous paper [36]. The final extract was analyzed using a gas chromatograph equipped with a high-resolution mass spectrometer (HRMS, HR-GS/MS; MStation-JMS700). The details of those procedures were also reported previously [37,38].

Dioxin concentrations are given as measured values and TEQ. The TEQ for each sample was calculated using the World Health Organization Toxic Equivalency Factors (WHO-TEF 2005) [30]. All congener concentrations and TEQs were determined in a lipid base.

### 3.4. Statistical Analysis

Data were summarized as median and interquartile range. Sister chromatid exchange frequency and dioxin concentrations were log-transformed to improve normality, and the Shapiro-Wilk test was applied to check normality. One-way analysis of variance, the Kruskal-Wallis test, and a chi-squared test were used to make comparisons between the three groups. The Tukey-Kreamer HSD test, the Wilcoxon rank sum test, and Fisher’s exact test with the Bonferroni correction were applied to compare each group individually. Homogeneity was assessed by the Levene test. The correlation between SCE frequency and dioxin concentration was examined using Spearman’s rank correlation coefficient. A multiple regression analysis was performed with SCE frequency as the dependent variable and categorical data for regional difference and each dioxin congener as the independent variables. A least-squares method was applied to the variables age, number of children, family income, occupation, and smoking status for the purpose of adjustment. JMP^®^ ver. 6.0 (SAS Institute, Cary, NC, USA) was used for statistical analysis. The significance level was set at *p* < 0.05.

### 3.5. Ethical Approvals

The purpose of this study was explained thoroughly, and written informed consent was obtained from each participant through their local people’s authorities committee. All data were transformed to codes in the analysis process for individuals and they were not identified. To conduct this survey, we obtained permission from the Medical Ethics Committee of Kanazawa University (permission no.: Health-89).

## 4. Conclusions

This study is the first time to examine the relationship between SCE frequency and dioxin congeners in Vietnam and shows that levels of hexa-, hepta-, and octaCDD are closely related to SCE frequency more than TCDD and that the level of dioxin (pg/g lipid) is potentially more useful as an indicator than TEQ value for explaining SCE frequency.

## Figures and Tables

**Table 1.** Characteristics of subjects from the high and moderate TCDD (tetrachlorodibenzo-p-dioxin) groups and the non sprayed group.

Itemes		High TCDD Group (1)		Moderate TCDD Group (2)		Non Sprayed Group (3)		*p* Value	Multiple Comparison		
			
		*n* = 21		*n* = 23		*n* = 19			1 *vs.* 2	1 *vs.* 3	2 *vs.* 3
Age [years]		33.0	(27.5–37.0)	31.0	(28.0–36.0)	30.0	(27.0–32.0)	0.441 a	n.s d	n.s d	n.s d
Height [cm]		150.2	(148.4–155.4)	149.9	(147.2–152.3)	151.2	(149.1–155.0)	0.102 b	n.s e	n.s e	n.s e
Weight [kg]		44.0	(43.0–46.0)	41.0	(40.0–47.0)	45.0	(42.0–48.0)	0.122 a	n.s d	n.s d	n.s d
BMI [kg/m^2^]		19.3	(18.3–20.6)	18.7	(17.7–20.9)	19.2	(18.3–20.5)	0.792 b	n.s e	n.s e	n.s e
Number of children		2.0	(1.0–2.5)	2.0	(2.0–3.0)	1.0	(1.0–2.0)	0.079 a	n.s d	n.s d	n.s d
Residence period [years]		27.0	(18.5–30.5)	30.0	(24.0–35.0)	28.0	(7.0–32.0)	0.187 a	n.s d	n.s d	n.s d
Family income [×10^4^ VND/month]		150	(100–180)	100	(70–150)	80	(50–120)	0.029 a	n.s d	*,^d^	n.s d

Occupation	Farmer	11	(52.4%)	22	(95.7%)	16	(84.2%)	0.002 c	**,^f^	n.s f	n.s f
	Other	10	(47.6%)	1	(4.3%)	3	(15.8%)	-	-	-	-

Smoking status	Smoker	0	(0.0%)	3	(13.0%)	0	(0.0%)	-	n.s f	-	n.s f
	Non smoker	21	(100.0%)	20	(87.0%)	19	(100.0%)	-	-	-	-

Data are shown as median (interquartile range) or number (%). BMI: Body mass index; VND: Vietnamese Dong;

a Kruskal-Wallis test;

b One-way ANOVA;

c Chi-squared test;

d Wilcoxon rank sum test with Bonferroni correction;

e Tukey-Kramer HSD test;

f Fisher’s exact test with Bonferroni correction;

n.s: not significant;

* *p* < 0.05;

** *p* < 0.01.

**Table 2.** Comparison of SCE (sister chromatid exchange), dioxin congeners and total concentrations in breast milk between high and moderate TCDD groups and non sprayed group.

Items	High TCDD Group (1)		Moderate TCDD Group (2)		Non Sprayed Group (3)		*p* Value	Multiple Comparison		
			
	*n* = 21		*n* = 23		*n* = 19			1 *vs.* 2	1 *vs.* 3	2 *vs.* 3
SCE [values/cell]	2.40	(2.28–2.72)	2.19	(2.00–2.35)	1.48	(1.35–1.71)	<0.001 a	*,^c^	***,^c^	***,^c^

*Dioxins [pg/g lipid]*										

2,3,7,8-TetraCDD	1.39	(1.15–2.07)	0.57	(0.31–0.74)	0.55	(0.48–0.66)	<0.001 a	***,^c^	***,^c^	n.s c
1,2,3,7,8-PentaCDD	3.62	(2.85–5.57)	1.68	(1.25–2.66)	1.25	(1.00–1.35)	<0.001 a	***,^c^	***,^c^	*,^c^
1,2,3,4,7,8-HexaCDD	2.83	(1.94–3.70)	1.11	(0.41–1.78)	0.50	(0.27–0.59)	<0.001 a	***,^c^	***,^c^	*,^c^
1,2,3,6,7,8-HexaCDD	9.05	(7.63–13.03)	4.87	(3.18–7.50)	1.20	(0.75–1.61)	<0.001 a	***,^c^	***,^c^	***,^c^
1,2,3,7,8,9-HexaCDD	2.53	(1.89–4.33)	1.01	(0.68–2.02)	0.48	(0.24–0.58)	<0.001 a	***,^c^	***,^c^	***,^c^
1,2,3,4,6,7,8-HeptaCDD	18.31	(13.53–26.91)	12.35	(7.31–16.35)	1.35	(1.19–1.58)	<0.001 a	**,^c^	***,^c^	***,^c^
OctaCDD	57.40	(45.39–85.08)	34.88	(21.09–59.02)	4.92	(4.20–6.73)	<0.001 a	*^c^	***,^c^	***,^c^

*Furans [pg/g lipid]*										

2,3,7,8-TetraCDF	0.60	(0.50–0.76)	0.51	(0.32–0.69)	1.01	(0.80–1.34)	<0.001 a	n.s c	**,^c^	***,^c^
1,2,3,7,8-PentaCDF	0.84	(0.52–1.55)	0.63	(0.28–1.01)	0.51	(0.40–0.62)	<0.001 a	n.s c	*,^c^	n.s c
2,3,4,7,8-PentaCDF	6.35	(5.01–11.17)	3.07	(2.16–5.15)	2.70	(1.69–3.39)	<0.001 a	***,^c^	***,^c^	n.s c
1,2,3,4,7,8-HexaCDF	17.84	(14.75–35.03)	9.87	(5.37–16.39)	1.58	(0.64–2.80)	<0.001 b	**,^d^	**,^d^	***,^d^
1,2,3,6,7,8-HexaCDF	9.70	(7.94–21.92)	5.54	(4.03–10.49)	0.95	(0.60–1.72)	<0.001 a	*,^c^	***,^c^	***,^c^
1,2,3,7,8,9-HexaCDF	0.43	(0.09–0.93)	0.29	(0.18–0.50)	0.07	(0.04–0.09)	<0.001 a	n.s c	**,^c^	***,^c^
2,3,4,6,7,8-HexaCDF	1.56	(1.13–2.67)	0.81	(0.35–1.52)	0.36	(0.23–0.43)	<0.001 a	**,^c^	***,^c^	**,^c^
1,2,3,4,6,7,8-HeptaCDF	13.71	(9.54–29.27)	9.26	(6.21–17.19)	0.88	(0.64–1.37)	<0.001 a	n.s c	***,^c^	***,^c^
1,2,3,4,7,8,9-HeptaCDF	2.31	(1.33–4.16)	1.37	(0.50–2.59)	0.08	(0.04–0.12)	<0.001 a	*,^c^	***,^c^	***,^c^
OctaCDF	0.21	(0.08–1.06)	0.22	(0.11–0.52)	0.07	(0.04–0.16)	0.004 a	n.s c	*,^c^	**,^c^

*Total [pg/g lipid]*										

PolyCDDs	107.80	(80.62–132.62)	58.38	(33.33–82.43)	10.34	(8.84–13.12)	<0.001 b	***,^d^	***,^d^	***,^d^
PolyCDFs	58.33	(43.97–102.50)	31.27	(21.71–59.26)	9.24	(4.94–12.70)	<0.001 a	**,^c^	***,^c^	***,^c^
PolyCDDs/Fs	165.82	(127.67–222.53)	98.69	(58.16–156.22)	20.97	(15.69–26.23)	<0.001 a	**,^c^	***,^c^	***,^c^

*Total-TEQ [pg-TEQ/g lipid]*										

PolyCDDs-TEQ	6.89	(5.62–9.92)	3.30	(2.24–4.52)	1.96	(1.69–2.34)	<0.001 a	**,^c^	***,^c^	***,^c^
PolyCDFs-TEQ	4.96	(4.19–9.81)	2.65	(1.68–4.34)	1.39	(0.75–1.76)	<0.001 a	***,^c^	***,^c^	***,^c^
PolyCDDs/Fs-TEQ	12.62	(10.00–17.11)	5.90	(4.00–9.25)	3.40	(2.51–4.10)	<0.001 a	***,^c^	***,^c^	***,^c^

Data are shown as median (interquartile range) and log-transformed. SCE: sister chromatid exchange; CDD: chlorinated dibenzodioxins; CDF: chlorinated dibenzofurans; TEQ: toxic equivalent;

a Kruskal-Wallis test;

b One-way ANOVA;

c Wilcoxon rank sum test with Bonferroni correction;

d Tukey-Kramer HSD test;

n.s: not significant;

* *p* < 0.05;

** *p* < 0.01;

*** *p* < 0.001.

**Table 3.** Relationship between SCE and dioxin congeners and total concentrations in breast milk (*n* = 63).

Dioxin Congers and Total Concentration	*R*	*p* Value
*Dioxins*		

2,3,7,8-TetraCDD	0.45	<0.001
1,2,3,7,8-PentaCDD	0.49	<0.001
1,2,3,4,7,8-HexaCDD	0.54	<0.001
1,2,3,6,7,8-HexaCDD	0.63	<0.001
1,2,3,7,8,9-HexaCDD	0.54	<0.001
1,2,3,4,6,7,8-HeptaCDD	0.58	<0.001
OctaCDD	0.61	<0.001

*Furans*		

2,3,7,8-TetraCDF	−0.42	<0.001
1,2,3,7,8-PentaCDF	0.04	0.782
2,3,4,7,8-PentaCDF	0.38	0.002
1,2,3,4,7,8-HexaCDF	0.55	<0.001
1,2,3,6,7,8-HexaCDF	0.55	<0.001
1,2,3,7,8,9-HexaCDF	0.20	0.110
2,3,4,6,7,8-HexaCDF	0.38	0.002
1,2,3,4,6,7,8-HeptaCDF	0.53	<0.001
1,2,3,4,7,8,9-HeptaCDF	0.56	<0.001
OctaCDF	0.19	0.138

*Total*		

PolyCDDs	0.62	<0.001
PolyCDFs	0.52	<0.001
PolyCDDs/Fs	0.57	<0.001

*Total-TEQ*		

PolyCDDs-TEQ	0.56	<0.001
PolyCDFs-TEQ	0.50	<0.001
PolyCDDs/Fs-TEQ	0.54	<0.001

Data are log-transformed. CDD: chlorinated dibenzodioxins; CDF: chlorinated dibenzofurans; TEQ: toxic equivalent; *R*: Spearman’s rank correlation coefficient.

**Table 4.** Relationship between SCE and regional difference as determined by multiple regression analysis (*n* = 63).

Model	Standardized *β*	*p* Value	Adjusted *R*^2^	*p* Value
*Model 1*				

Sprayed area a	0.73	<0.001	0.49	<0.001

*Model 2*				

High TCDD group b	0.30	0.011	0.54	<0.001
Non sprayed group b	−0.58	<0.001	-	-

SCE is log-transformed.

a Non sprayed group and

b moderate TCDD group taken as reference category.

Models 1 and 2 were adjusted for age, number of children, family income, occupation (farmer/other), and smoking status (smoker/non smoker).

**Table 5.** Relationship between SCE and each dioxin congeners and total concentration in breast milk as determined by multiple regression analysis (*n* = 63).

Dioxin Congers and Total Concentration	Standardized *β*	*p* Value	Adjusted *R*^2^	*p* Value
*Dioxins*				

2,3,7,8-TetraCDD	0.34	0.016	0.09	0.076
1,2,3,7,8-PentaCDD	0.42	0.001	0.16	0.014
1,2,3,4,7,8-HexaCDD	0.40	0.002	0.15	0.020
1,2,3,6,7,8-HexaCDD	0.60	<0.001	0.35	<0.001
1,2,3,7,8,9-HexaCDD	0.48	<0.001	0.23	0.002
1,2,3,4,6,7,8-HeptaCDD	0.64	<0.001	0.39	<0.001
OctaCDD	0.65	<0.001	0.37	<0.001

*Furans*				

2,3,7,8-TetraCDF	−0.42	0.002	0.14	0.021
1,2,3,7,8-PentaCDF	−0.00	0.974	−0.00	0.503
2,3,4,7,8-PentaCDF	0.33	0.011	0.10	0.061
1,2,3,4,7,8-HexaCDF	0.51	<0.001	0.26	<0.001
1,2,3,6,7,8-HexaCDF	0.51	<0.001	0.26	<0.001
1,2,3,7,8,9-HexaCDF	0.10	0.435	0.00	0.428
2,3,4,6,7,8-HexaCDF	0.27	0.040	0.06	0.136
1,2,3,4,6,7,8-HeptaCDF	0.54	<0.001	0.29	<0.001
1,2,3,4,7,8,9-HeptaCDF	0.58	<0.001	0.32	<0.001
OctaCDF	0.09	0.529	−0.00	0.453

*Total*				

PolyCDDs	0.64	<0.001	0.38	<0.001
PolyCDFs	0.47	<0.001	0.22	0.003
PolyCDDs/Fs	0.59	<0.001	0.32	<0.001

*Total-TEQ*				

PolyCDDs-TEQ	0.50	<0.001	0.23	0.002
PolyCDFs-TEQ	0.42	<0.001	0.17	0.001
PolyCDDs/Fs-TEQ	0.48	<0.001	0.21	0.003

SCE and dioxin concentrations are log-transformed. CDD: chlorinated dibenzodioxins; CDF: chlorinated dibenzofurans. All models are adjusted for age, number of children, family income, occupation (farmer/other), and smoking status (smoker/non smoker).
